# Callose and Salicylic Acid Are Key Determinants of Strigolactone-Mediated Disease Resistance in Arabidopsis

**DOI:** 10.3390/plants13192766

**Published:** 2024-10-02

**Authors:** Xiaosheng Zhao, Qiuping Liu, Leitao Tan

**Affiliations:** School of Life Sciences, Guizhou Normal University, Guiyang 550025, China; zxs718@126.com (X.Z.); liuqiuping0999@163.com (Q.L.)

**Keywords:** strigolactones, callose, salicylic acid, disease resistance, Arabidopsis

## Abstract

Research has demonstrated that strigolactones (SLs) mediate plant disease resistance; however, the basal mechanism is unclear. Here, we provide key genetic evidence supporting how SLs mediate plant disease resistance. Exogenous application of the SL analog, *rac*-GR24, increased *Arabidopsis thaliana* resistance to virulent *Pseudomonas syringae*. SL-biosynthetic mutants and overexpression lines of more axillary growth 1 (*MAX1*, an SL-biosynthetic gene) enhanced and reduced bacterial susceptibility, respectively. In addition, *rac*-GR24 promoted bacterial pattern flg22-induced callose deposition and hydrogen peroxide production. SL-biosynthetic mutants displayed reduced callose deposition but not hydrogen peroxide production under flg22 treatment. Moreover, *rac*-GR24 did not affect avirulent effector-induced cell death between Col-0 and SL-biosynthetic mutants. Furthermore, *rac*-GR24 increased the free salicylic acid (SA) content and significantly promoted the expression of pathogenesis-related gene 1 related to SA signaling. Importantly, *rac*-GR24- and *MAX1*-induced bacterial resistance disappeared completely in Arabidopsis plants lacking both callose synthase and SA. Taken together, our data revealed that callose and SA are two important determinants in SL-mediated plant disease resistance, at least in Arabidopsis.

## 1. Introduction

Strigolactones (SLs) are a class of structurally diverse carotenoid-derived plant hormones [1,2]. As signal molecules, SLs are involved in communication between host plants and symbiotic/parasitic organisms, modulation of the plant shoot and root architecture, and plant adaptation to abiotic stress and nutrient deficiency [3,4]. In the model plant *Arabidopsis thaliana*, SL-biosynthetic mutants, *more axillary growth 3* (*max3*), *max4*, and *max1*, and SL-signaling mutants, *max2* and *DWARF 14* (*d14*), have been successfully identified. In detail, *max3* and *max4* have mutations in carotenoid cleavage dioxygenase7 (CCD7) and CCD8 [5,6], respectively. Correspondingly, *max1*, *max2*, and *d14* have mutations in a cytochrome P450 enzyme [7], the F-box protein MAX2 [8], and the α/β-hydrolase superfamily protein DWARF14 [9], respectively. In the field of botany, allelic mutants of SL biosynthesis/signaling genes and the bioactive SL analog *rac*-GR24 have been used extensively to characterize the biological role of SLs.

Plants have developed a series of systems to protect them from pathogen attacks. The innate immune response and hormone-dependent resistance are the best-characterized defense strategies. Plant immunity comprises pattern-triggered immunity (PTI) and effector-triggered immunity (ETI) [10,11,12]. PTI is the collective name for many pattern-induced responses, including callose (a β-1,3-glucan) deposition, reactive oxygen species (ROS) burst, activation of mitogen-activated protein kinases, and the expression of defense-related genes. By contrast, ETI is based on the hypersensitive response (HR) induced by pathogen effectors, a form of plant-programmed cell death. Salicylic acid (SA), jasmonic acid (JA), and ethylene (ET) are the best-known defense-related hormones [10,13]. Among them, SA is mainly responsible for resistance to biotrophic and hemi-biotrophic pathogens, JA/ET is responsible for resistance to necrotrophic pathogens, and SA and JA/ET antagonize each other. Generally, PTI, ETI, and defense-related hormone signaling are the important indicators that characterize the basic mechanism of plant disease resistance. 

Increasing evidence indicates that SLs positively regulate plant disease resistance [14,15]. For example, SL-deficient tomato (*Solanum lycopersicum*) plants were more susceptible to the necrotrophic fungi *Botrytis cinerea* and *Alternaria alternata* than the wild-type plants [16]. The exogenous application of *rac*-GR24 reduced symptom development caused by the biotrophic actinomycete *Rhodococcus fascians* in Arabidopsis, while SL-biosynthetic and signaling mutants were hypersensitive [17]. The *A. thaliana max2* mutant was more susceptible to the hemi-biotrophic bacterium *Pseudomonas syringae* and the necrotrophic bacterium *Pectobacterium carotovorum* [18]. In moss (*Physcomitrella patens*), knockout mutants of *CCD7* and *CCD8* were more susceptible to the phytopathogenic fungi *Sclerotinia sclerotiorum*, *Irpex* sp., and *Fusarium oxysporum* compared with wild-type plants [19]. In addition, rice (*Oryza sativa*) SL-biosynthetic (*d17*) or signaling (*d14*) mutants were hypersusceptible to the fungal pathogen *Magnaporthe oryzae* [20]. Recently, it was reported that SLs regulate SA-mediated *A. thaliana* resistance to *P. syringae* [21].

Currently, the mechanisms by which SLs regulate plant disease resistance are incompletely understood. In this study, we aimed to investigate the potential roles of SLs in plant disease resistance using the model system of *A. thaliana-P. syringae* interaction. We hypothesized that SLs contribute to plant disease resistance depending on the activation of PTI, ETI, and defense-related hormone signaling pathways in Arabidopsis. This study could uncover the mechanisms by which SLs control plant disease resistance and further improve our understanding of the biological roles of SLs.

## 2. Results

### 2.1. SLs Contribute to A. thaliana Resistance to P. syringae 

**Figure 1:**
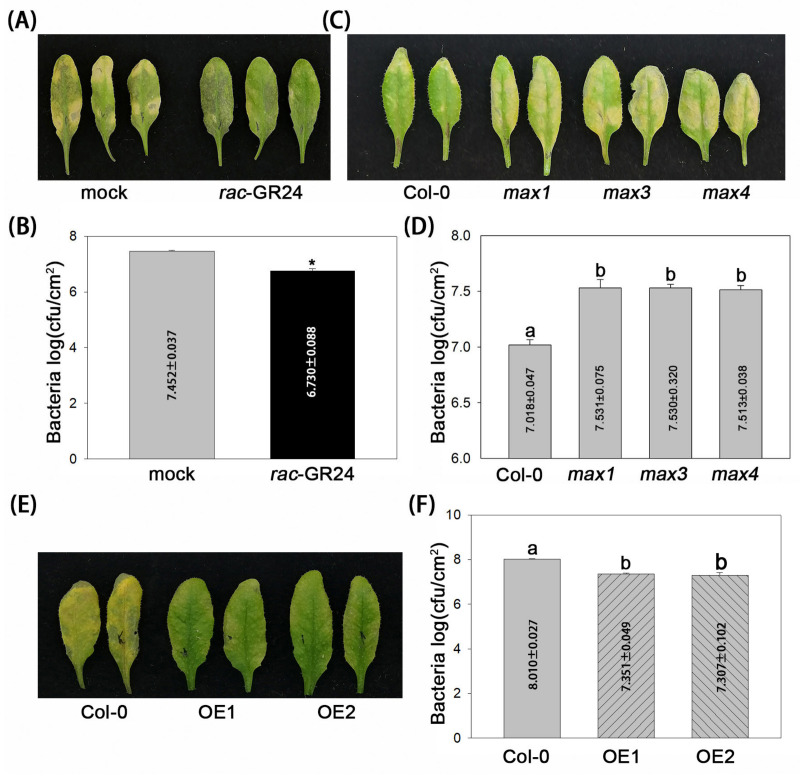
SLs enhance *A. thaliana* resistance to virulent *P. syringae*. (**A**,**B**) Disease symptoms and bacterial growth in *rac*-GR24-treated Col-0 plants. Leaves were sprayed with *rac*-GR24 or DMSO (as a mock control). (**C**,**D**) Disease symptoms and bacterial growth in SL biosynthesis mutants. (**E**,**F**) Disease symptoms and bacterial growth in two *MAX1*-transgenic lines (in Col-0), OE1 and OE2. Health leaves were inoculated with *Pseudomonas syringae* pv. *tomato* DC3000. Photographs were taken, and bacterial numbers were assessed at 3 dpi. Error bars show the mean ± SD. *, *t*-test, *p* < 0.05. Different letters indicate values that are significantly different (*p* < 0.05) from each other as determined by one-way ANOVA (SPSS v19.0).

The *A. thaliana-P. syringae* pathosystem was used to assess the role of the SL analog *rac*-GR24 in plant disease resistance. Columbia-0 (Col-0) plants were pretreated with *rac*-GR24 (1 µM) and DMSO (as a mock control) for 4 h, respectively, before inoculation with the virulent *P. syringae* pv. *tomato* strain DC3000. Compared with DMSO treatment, *rac*-GR24 only induced slight chlorosis in infected leaves at 3 dpi (Figure 1A). Correspondingly, bacterial titers under *rac*-GR24 treatment were approximately 5-fold reduced compared with those under DMSO treatment (Figure 1B). These results indicated that *rac*-GR24 improves *A. thaliana* resistance to DC3000.

Next, we conducted pathogenicity analysis using SL biosynthesis mutants, including *max1*, *max3*, and *max4*. Compared with Col-0 (as a control), DC3000 infected leaves on the mutant plants exhibited severe chlorosis (Figure 1C), and bacterial titers were approximately 3-fold higher compared with that of the control (Figure 1D). These results indicated that SLs negatively regulate *A. thaliana* susceptibility to DC3000. To provide the reverse proof, we generated *MAX1*-transgenic Col-0 lines. Transgenic *MAX1* overexpressing lines OE1 and OE2 expressed high levels of the GFP-MAX1 mRNA and protein (Figure S1A); however, there was no apparent effect on the growth of Col-0 (Figure S1B). Pathogenicity analysis of OE1 and OE2 showed that these lines displayed only slight chlorosis (Figure 1E) and had an approximately 5-fold reduction in the DC3000 titer (Figure 1F) compared with Col-0 plants only expressing GFP. Although the SL levels in the transgenic lines were not determined, the results of *MAX1* overexpression provided key support for the hypothesis that endogenous SLs regulate disease resistance. These results confirmed that endogenous SLs have a crucial role in promoting *A. thaliana* resistance to DC3000.

To exclude the potential influence of SLs on DC3000 multiplication, bacterial growth was assessed on KB agar medium containing various concentrations of *rac*-GR24 (0, 0.25, 1.0, 2.5, and 10 µM). After 72 h of culture, all colonies appeared round and glossy under different concentrations of *rac*-GR24, and the colony diameter at each concentration of *rac*-GR24 was similar to that of the parallel control (Figure S2). These results indicated the non-toxicity of *rac*-GR24 toward DC3000 multiplication. Therefore, we deduced that SLs probably regulate disease resistance by manipulating defense-related events.

### 2.2. SLs Enhance Bacterial Pattern-Induced Callose Deposition

**Figure 2:**
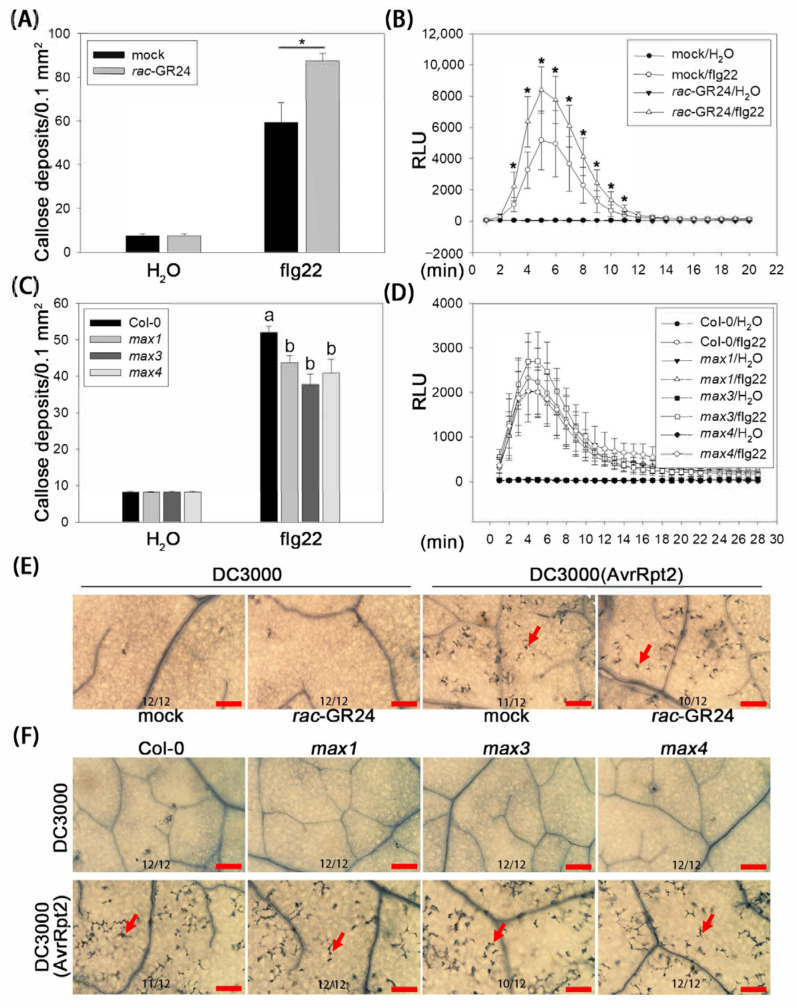
SLs increase bacterial pattern-induced callose deposition. (**A**,**B**) Flagellin peptide flg22-induced callose deposition and H_2_O_2_ production in *rac*-GR24-treated Col-0 plants. Leaves were sprayed with *rac*-GR24 or DMSO (as a mock control). (**C**,**D**) flg22-induced callose deposition and H_2_O_2_ production in SL biosynthesis mutants. The results shown are representative of three independent experiments. Each data point consists of eight replicates. Error bars show the mean ± SD. *, *t*-test, *p* < 0.05. Different letters indicate values that are significantly different (*p* < 0.05) from each other as determined by one-way ANOVA (SPSS v19.0). (**E**,**F**) AvrRpt2-induced cell death in *rac*-GR24-treated Col-0 plants and SL biosynthesis mutants. Col-0 plants were treated with *rac*-GR24 and DMSO (as a mock control). Leaves were syringe-infiltrated with DC3000 or DC3000 (AvrRpt2). Leaves (n = 12) stained using aniline blue and trypan-blue were analyzed under a microscope. The red arrows indicate dots of cell death. Scale bar = 150 μm.

To understand the effects of SLs on defense-related immune events, we first analyzed bacterial pattern-induced callose deposition (associated with cell wall enhancement) and the ROS burst in Arabidopsis. Col-0 plants were pretreated with *rac*-GR24 or DMSO. Compared with DMSO treatment, *rac*-GR24 treatment increased the bacterial flagellin peptide flg22-induced callose fluorescence by 1.48-fold (Figure 2A and Figure S3A) and increased flg22-induced hydrogen peroxide (H_2_O_2_) levels (Figure 2B). The flg22-induced cell wall enhancement and ROS burst were further analyzed in SL-biosynthetic mutants. Compared with Col-0 plants, callose fluorescence in *max1*, *max3*, and *max4* was reduced by 16.1–31.2% (Figure 2C and Figure S3B). However, there was a difference in H_2_O_2_ production between Col-0 and the SL-biosynthetic mutants (Figure 2D). These results suggested that SLs promote PTI, at least by enhancing bacterial pattern-induced callose deposition.

Next, we examined avirulent effector-induced HR (Hypersensitive Response). Col-0 plants were pretreated with *rac*-GR24 (1 µM) or DMSO before inoculation with the bacterium DC3000 (AvrRpt2), which secretes the avirulent effector protein, AvrRpt2. Trypan-blue staining showed that neither *rac*-GR24 nor DMSO treatment induced HR on leaves in the presence of DC3000 (Figure 2E). Under DC3000 (AvrRpt2) inoculation, HR was observed on all infected leaves; however, there was no observed difference in HR between the *rac*-GR24- and DMSO-treated leaves (Figure 2E). In addition, there was no apparent difference in DC3000 (AvrRpt2)-induced HR among *max1*, *max3*, *max4*, and Col-0 plants (Figure 2F). Taken together, these results implied that callose deposition might be an important immune event in SL-induced improvement of *A. thaliana* resistance.

### 2.3. rac-GR24 Increases Salicylic Acid Signaling

**Figure 3:**
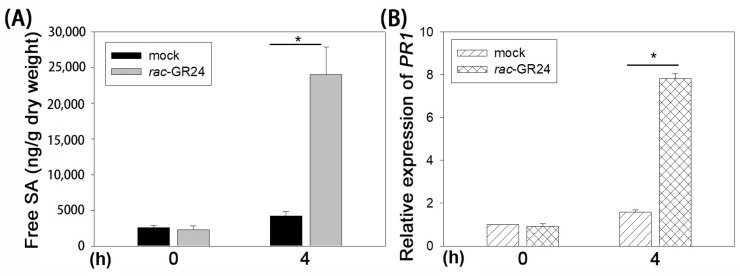
*rac*-GR24 activates the SA pathway. (**A**) *rac*-GR24 induces the accumulation of the free SA content. Col-0 plants were treated with *rac*-GR24 and DMSO (as a mock control). The free SA content of leaves was measured. (**B**) *rac*-GR24 promotes SA-dependent expression of PR1. Error bars show the mean ± SD. *, *t*-test, *p* < 0.01.

DC3000 is a hemi-biotrophic pathogen; therefore, we examined whether SLs affect SA biosynthesis and signaling. The results showed that the free SA content in Col-0 plants under *rac*-GR24 (1 µM) treatment for 4 h increased by approximately 6-fold compared with that in plants under DMSO treatment (Figure 3A). In parallel with the free SA content, the expression of PR1, an SA-dependent pathogenesis-related protein 1 gene, increased by approximately 5-fold under *rac*-GR24 (1 µM) treatment (Figure 3B). These results implied that the SA pathway might also be a crucial factor in SL-mediated *A. thaliana* resistance.

### 2.4. Callose and SA Are Required for SL-Induced Resistance to P. syringae

**Figure 4:**
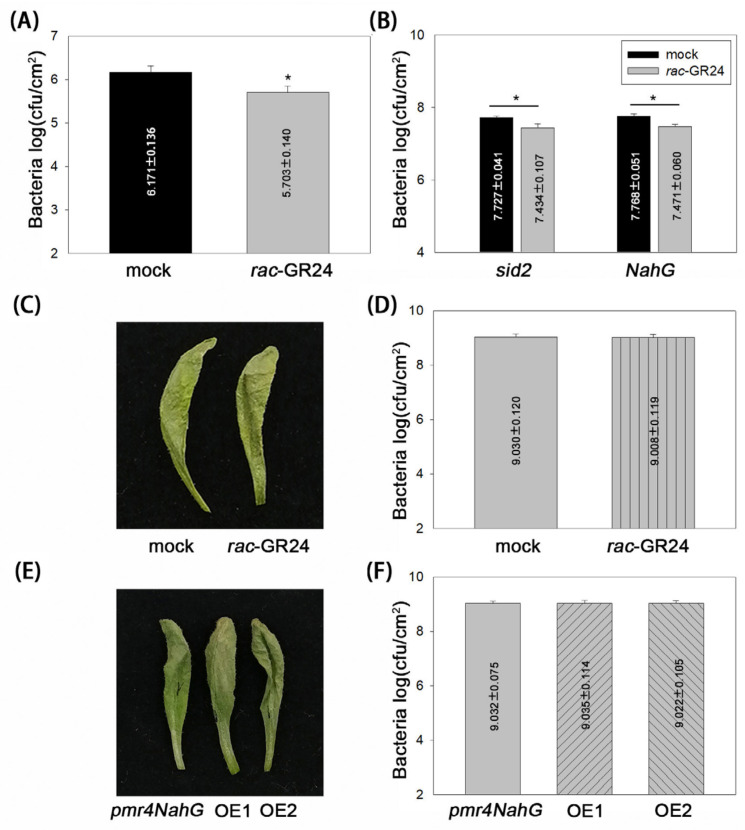
SL-induced *A. thaliana* resistance to *P. syringae* depends on callose and SA. (**A**) Bacterial growth in *rac*-GR24-treated *pmr4* plants. (**B**) Bacterial growth in *rac*-GR24-treated *sid2* and NahG-transgenic plants. (**C**,**D**) Disease symptoms and bacterial growth in *rac*-GR24-treated *pmr4*NahG plants. Leaves were treated with *rac*-GR24 and DMSO (as a mock control). (**E**,**F**) Disease symptoms and bacterial growth in two *MAX1*-transgenic lines (in *pmr4*-NahG), oe1 and oe2. Leaves were inoculated with DC3000. Photographs were taken, and bacterial numbers were assessed at 3 dpi. Error bars show the mean ± SD. *, *t*-test, *p* < 0.05.

We next detected whether callose and SA are required for SL-mediated disease resistance. To investigate the requirement for callose, the *A. thaliana* mutant *pmr4*, which produces dramatically less pathogen-induced callose deposition, was used for the pathogenicity analysis. At 3 dpi, the DC3000 titers in *pmr4* were reduced by approximately 3-fold under *rac*-GR24 (1 µM) treatment compared with that under DMSO treatment (Figure 4A). 

To determine the role of SA, the SA-deficient *sid2* mutant and the SA-deficient NahG-transgenic Arabidopsis line were used for the pathogenicity analysis. At 3 dpi, the DC3000 titers in both *sid2* plants and the NahG-transgenic line decreased by approximately 2-fold under *rac*-GR24 (1 µM) treatment compared with that under DMSO treatment (Figure 4B). Considering the approximately 5-fold reduction of DC3000 titers in Col-0 plants treated with *rac*-GR24 (Figure 1A), the decreased reduction in the bacterial titers in *pmr4*, *sid2*, and the NahG-transgenic line indicated that SL-mediated disease resistance depends on callose and SA.

To further test whether callose and SA are key determinants for SL-mediated disease resistance, we performed the pathogenicity analysis using *pmr4*-NahG plants, a line formed by crossing *pmr4* with the NahG-transgenic plant. Interestingly, regardless of *rac*-GR24 or DMSO treatment, severe necrosis was displayed in DC3000-infected leaves at 3 dpi (Figure 4C), and no difference in bacterial titers was detected between the two treatments (Figure 4D). In addition, we generated the CaMV 35S promoter-controlled *MAX1* transgenic lines on the *pmr4* NahG background. Transgenic lines oe1 and oe2 had high levels of the GFP MAX1 fusion protein and mRNA (Figure S4A), which had no observable impact on the growth of *pmr*-4NahG (Figure S4B). The pathogenicity analysis results showed that oe1, oe2, and *pmr4*-NahG only expressing GFP all exhibited severe necrosis in DC3000-infected leaves at 3 dpi (Figure 4E), and no significant difference in DC3000 titers was observed between the transgenic lines and *pmr4*-NahG plants expressing GFP (Figure 4F). These results confirmed the dominant function of callose and SA in SL-mediated resistance to DC3000.

Although JA and ET are the main plant hormones that defend against necrotrophic pathogens, we explored their effects on SL function during the *A. thaliana-P. syringae* interaction. The JA-signaling mutant *coi1* and the ET-signaling mutant *ein2* were subjected to pathogenicity tests. The results showed that *rac*-GR24 still induced an approximately 5-fold reduction in DC3000 titers in both *coi1* and *ein2* plants (Figure S5A). This similar reduction of bacterial titers to that in Col-0 plants treated with *rac*-GR24 (Figure 1A) indicated that the JA and ET signaling are not required for SL-mediated resistance to DC3000.To better understand the role of EIN2, we detected the contents of SA and the expression of PR1 in *rac*-GR24-treated *ein2* mutant plants. Compared with DMSO treatment, *rac*-GR24 (1 µM) induced an approximately 7.5-fold increase in free SA (Figure S5B) and an approximately 2.6-fold increase in *PR1* expression (Figure S5C), indicating the response of *ein2* to *rac*-GR24.

## 3. Discussion

To date, it has been demonstrated that SLs contribute to disease resistance in certain plants, including tomato [16], rice [20], moss [19], and Arabidopsis [17,18,22]. Despite SLs mediating resistance only to specific pathogens [14], they still have potential value in the field of plant protection. *Arabidopsis thaliana* is one of the most important materials for studying the molecular mechanism and basis of plant disease resistance. In this report, we assessed the role of SLs during the *A. thaliana-P. syringae* interaction examined the effect of SLs on innate immunity and SA pathways and analyzed the necessity of several defense-related signaling pathways for SL function. The findings of the present study revealed that callose and SA are required for SL-enhanced plant disease resistance, at least in *A. thaliana* defense against pathogenic *P. syringae*.

Previous reports have suggested that the SL-insensitive *max2* mutant is susceptible to the pathogenic bacterium DC3000 [18]. This begs the question as to whether SLs affect *A. thaliana* resistance to DC3000. MAX2 also functions as a key regulator in signal transduction of karrikins (KARs), a class of plant-derived small molecules [23]; however, whether KARs influence *A. thaliana* resistance is unknown. Our data demonstrated that the exogenous application of the SL analog *rac*-GR24 increased disease resistance to DC3000 in Col-0 plants. Similar to a recent report [22], SL-biosynthetic mutants *max1*, *max3*, and *max4* were more susceptible to DC3000 than Col-0 (Figure 1). However, it should be pointed out that no differences were observed in the growth of DC3000 between KAR1 (dissolved in DMSO) and DMSO treatment at 3 dpi (Figure S6). These results confirmed that SLs, but not KAR1, regulate *A. thaliana* resistance to DC30000.

As SL biosynthesis-related genes, the homologs of *MAX1* have been studied extensively using transgenic plants. For example, *Liriodendron chinense LcMAX1*-transgenic Arabidopsis lines [24] showed no phenotypic differences compared with wild-type plants. Overexpressing the *Brachypodium distachyon BdCYP711A29* gene did not affect the development of *B. distachyon* [25]. Consistent with these studies, our data showed that overexpression of *GFP-MAX1* did not alter the growth of transgenic Arabidopsis plants, including Col-0 (Figure S1B) and *pmr4*-NahG (Figure S4B). These results implied the functional conservation of MAX1 homologs. In particular, *LcMAX1* is involved in SL biosynthesis in *L. chinense* [24] and overexpressing *BdCYP711A29* in *B. distachyon*-mediated resistance to the fungus *Fusarium graminearum* by increasing the biosynthesis of the SL orobanchol [25], directly indicating the close relationship between MAX1 homologs and SL biosynthesis. Considering the bacterial susceptibility of the *max1* mutant (Figure 1), *GFP-MAX1* overexpression-enhanced bacterial resistance most likely resulted from increased endogenous SLs, although the SL levels in the transgenic lines remain unknown.

Plants employ a two-branched innate immunity, PTI and ETI, to efficiently defend against most pathogens [11,12]. Our data revealed that *rac*-GR24 enhanced bacterial pattern flg22-induced callose deposition (Figure 2). In addition, SL-biosynthetic mutants *max1*, *max3*, and *max4* showed a reduction in flg22-induced callose deposition compared with Col-0 (Figure 2), indicating that callose might be an important target of SLs to enhance plant disease resistance. In support of this, a previously published comparative transcriptome analysis revealed that many genes potentially involved in cellulose synthesis, such as genes encoding cellulose synthase family A, C, and E, and genes involved in cell wall modification, such as those encoding xyloglucan endo transglycosylase/hydrolases (XTHs) and expansins (EXPs), were downregulated in the rice SL-signaling *d14* mutant under *M. oryzae* infection [20]. 

Our data showed only demonstrated an effect of *rac*-GR24, but not SL-biosynthetic mutants, on flg22-induced H_2_O_2_ generation (Figure 2). This result indicated that *rac*-GR24 and endogenous SLs might function differentially during the oxidative burst. However, they showed functionally consistent effects on flg22-induced callose deposition. This was not surprising because the flg22-triggered callose deposition in the *det3* and *ost2-1D* mutants was indistinguishable from that in wild-type plants, although flg22 induced an oxidative burst and MAPK activation [26]. In addition, H_2_O_2_ levels were remarkably lower in the rice *d14* mutant and the SL-biosynthetic mutant *d17* than in the wild-type during *M. oryzae* infection [20], which partly contradicts our result that SLs had no effects on the flg22-induced oxidative burst (Figure 2). We deduced that this might have resulted from different hosts responding to different pathogens and/or patterns.

SA is the best-characterized hormone related to plant disease resistance [10]; however, the relationship between SLs and SA is complicated. As reported previously, the SL-deficient tomato mutant *Slccd8* showed a reduction of approximately 50% in the levels of SA compared with the wild-type, whereas the expression of PR1a was not altered in Slccd8 [16]. Compared with wild-type plants, the 4-week-old *max2* mutant plants had higher SA levels and the expression of the SA marker gene *PR1* after DC3000 inoculation [18]. Moreover, the application of 10 µM *rac*-GR24 (with 0.02% DMSO as control) failed to alter the expression of *PR1* in 10-day-old Col-0 seedlings [22]. SA accumulation and the expression of *PR1* were not influenced by 10 µM *rac*-GR24 (with 0.02% acetone as control) in 3-week-old Col-0 plants, while *PR1* expression, but not SA contents, was upregulated upon DC3000 inoculation [21]. Furthermore, our data showed that 1 µM *rac*-GR24 (with 0.7 mM DMSO as a control) increased free SA accumulation and the expression of *PR1* in 4 to 5-week-old Col-0 plants (Figure 3). Similarly, 1 µM *rac*-GR24 (with acetone as a mock control) significantly improved the production of SA in 10-day-old Col-0 seedlings [27]. Taken together, these findings indicated that SLs function differentially, which might be related to plant species and mutants, the dose of *rac*-GR24, or the different solvents used. Most likely, the ability of *rac*-GR24 to induce SA biosynthesis and signaling might be highlighted under the relatively high concentration of the solvent.

Our data showed that JA/ET signaling is not required for rac-GR24-mediated bacterial resistance (Figure 4). In support of this, we detected that SA biosynthesis and signaling could be induced by 1 µM *rac*-GR24 (Figure S5), implying the role of SA in *rac*-GR24-mediated disease resistance in the *ein2* mutant. Interestingly, Kusajima et al. [21] reported that ET signaling is involved in *rac*-GR24-induced disease resistance. Considering the differential action of *rac*-GR24 on the regulation of SA biosynthesis and signaling in Col-0 in the present study (Figure 3) and Kusajima’s report, we hypothesized that the dose of *rac*-GR24 and the concentration of solvent might lead to the distinct results of ET signaling in *rac*-GR24 mediated disease resistance.

## 4. Materials and Methods

### 4.1. Bacterial Strains, Plant Materials, and Growth Conditions

The bacterial strains included *Pseudomonas syringae* pv. *tomato* DC3000, DC3000 (AvrRpt2), *Agrobacterium tumefaciens* GV3101, and *Escherichia coli* DH5α. DC3000 and DC3000 (AvrRpt2) were grown at 28 °C in King’s B (KB) medium [28]. GV3101 was grown at 28 °C in Luria-Bertani (LB) medium [29]. DH5α was grown at 37 °C in LB medium. Selective media contained kanamycin at a final concentration of 25 μg/mL. *Arabidopsis thaliana* plants, including Col-0, *coi1* (*coi1-1*, [30]), *ein2* (*ein2-1*, [31]), *sid2* (sid2-2, [31]), the NahG-transgenic line ([32]), *pmr4* (CS3858, [33]), *pmr4*-NahG (CS67159, [33]), *max1* (CS9564, [8]), *max3* (CS9567, [5]), and *max4* (CS9567, [34]), were all of the Columbia ecotype. Seeds of *max1*, *max3*, *max4*, and *pmr4* mutants and the *pmr4*-NahG line were obtained from the Nottingham Arabidopsis Stock Centre (Nottingham, UK) and were genotyped using genotyping PCR primers (Table S1). All plants were grown in a growth room at 22 °C with a 12-h photoperiod and 65–70% relative humidity.

### 4.2. Hormone Treatment and Pathogenicity Analysis

Both Karrikin1 (KAR1) (Roche, Basel, Switzerland) and *rac*-GR24 (Chiralix, Nijmegen, The Netherlands) were separately dissolved with 0.7 M DMSO (Sigma, St. Louis, MO, USA) to a final concentration of 1 mM. To test *rac*-GR24 or KAR1, 4 to 5-week-old Arabidopsis plants were sprayed with 1 µM *rac*-GR24 or KAR1 and 0.7 mM DMSO (as a mock control), respectively, until the fog drops evenly cover the whole blade surface. After 4 h, healthy leaves of the Arabidopsis plants were syringe-inoculated with a suspension of approximately 10^6^ colony-forming units (cfu)/mL (OD600 = 0.002) of DC3000 until the whole blade was filled. To perform pathogenicity analysis on *MAX1* transgenic plants, healthy leaves of 4 to 5-week-old transgenes were syringe-inoculated as the above. The DC3000 strain was grown overnight, washed five times, and resuspended in 10 mM MgCl_2_ solution. At 3 days post-infection (dpi), photographs of leaves were obtained, and 12 leaf discs (three leaf discs per plant from four plants, 0.74 cm^2^ per disc, with every two discs as a sample, for a total of six samples) under each treatment condition were ground in 10 mM MgCl_2_ solution in a mortar. Dilutions of leaf homogenates were spotted onto KB agar medium and cultured for 48 h at 28 °C for bacterial counting. These experiments were repeated at least three times.

### 4.3. Bacterial Growth under rac-GR24 Treatment

The SL analog *rac*-GR24 was dissolved in 0.7 M DMSO (Sigma) to a final concentration of 1 mM. The DC3000 strain was grown overnight at 28 °C and then diluted with KB liquid medium to OD600 = 1.0. The bacterial dilution was dropped onto KB agar medium embedded with 0, 0.25, 1, 2.5, or 10 µM *rac*-GR24, respectively. In the corresponding control, the KB agar medium contained 0, 0.175, 0.7, 1.75, or 7 mM DMSO. After 72 h of culture at 28 °C, colony diameters were measured, and photographs were taken.

### 4.4. Vector Construction

The Arabidopsis full-length *MAX1* coding sequence was amplified by PCR using the primers listed in Table S1. The harvested amplicons were cloned into the binary vector pEGAD (with Basta resistance) to generate pEGAD-*MAX1*. The expression of *GFP-MAX1* was controlled by the CaMV 35S promoter. The constructed Pegad-MAX1 vector and the pEGAD empty vector were transformed into *A. tumefaciens* GV3101, separately.

### 4.5. Generation of Transgenic Plants and Western Blotting

The above vectors were separately transformed into Col-0 plants by dipping flowers in a GV3101 suspension (OD600 = 0.5) diluted with 5% sucrose solution containing 0.01% SILWET^®^ L-77 (GE healthcare Bio-Sciences AB, Uppsala, Sweden). The Arabidopsis transformants were grown in plant growth bowls. Ten-day-old seedlings were sprayed with 25 µg/mL glufosinate ammonium until the fog drops evenly covered the whole plant. The total proteins of the leaves of the transgenic lines were extracted using a protein-extraction buffer. The GFP-MAX1 fusion protein was detected among the total proteins using anti-GFP antibodies (Roche). The expression of *GFP-MAX1* was further assessed using PCR with cDNA from the transgenic lines as templates. The primers used are listed in Table S1.

### 4.6. Aniline Blue Staining and Fluorescence Observation

Healthy leaves of 4 to 5-week-old Arabidopsis plants were syringe-infiltrated with 1 μM flg22, a 22 amino acid bacterial flagellin peptide [35], dissolved in distilled water. At 24 h post-infection (hpi), infiltrated leaves were harvested and stained with aniline blue solution [36]. Callose fluorescence was observed using a fluorescence microscope (Nikon, Tokyo, Japan) with a 10× objective under ultraviolet light. The fluorescence intensity of the callose in each photograph was measured using Image J software (Image J 1.54, NIH, Bethesda, MD, USA).

### 4.7. Detection of the H_2_O_2_

Healthy leaves of 4 to 5-week-old Arabidopsis plants were sliced into approximately 1 mm strips. The sliced leaves were placed into 96-well plates containing 200 µL of distilled H_2_O and incubated overnight under weak light. After discarding the distilled H_2_O, a solution containing 1 µM flg22, 20 mM luminol (Sigma), and 1 µg of horseradish peroxidase (Sigma) was added to the wells of the 96-well plates. Luminescence was assessed immediately using a Luminometer (Promega, Madison, WI, USA).

### 4.8. Trypan-Blue Staining and Microscopy

Healthy leaves of 4 to 5-week-old Arabidopsis plants were syringe-inoculated with a 10^6^ cfu/mL suspension of DC3000 (AvrRpt2). At 22 hpi, infected leaves were stained overnight with trypan-blue solution [37]. The stained leaves were destained with saturated chloral hydrate solution. Completely decolorized leaves were observed under a microscope (Nikon) with a 10× objective under normal light.

### 4.9. SA Measurements

Four to 5-week-old Arabidopsis plants were sprayed with 1 µM *rac*-GR24 (with 0.7 mM DMSO as a mock control). After 4 h, treated leaves were harvested and freeze-dried, and 25 mg of each freeze-dried material was powdered in liquid nitrogen. Then, the powder was homogenized with 1 mL of MeOH: H_2_O (0.01% HCOOH) (10:90) and shaken for 3 min on a vortex oscillator. The supernatant was collected into a new tube after centrifugation (10,000× *g*, 15 min). Salicylic acid-d5 (SA-d5) was added to each sample as the internal standard. Free SA contents were determined using a high-performance liquid chromatography-tandem mass spectrometry (HPLC-MS/MS) system. The instrumentation and the conditions used in this chromatographic analysis were the same as reported previously [31].

### 4.10. Quantitative Real-Time Reverse Transcription PCR (qRT-PCR) Analysis

The total RNA of 4 to 5-week-old healthy Arabidopsis leaves was extracted using an RNeasy Plant Mini kit (Qiagen, Hilden, Germany). RNA samples were digested with DNase Turbo DNAfree (Promega). Then, 1 µg RNA of each sample was subjected to reverse transcription with SuperScript III reverse transcriptase (Invitrogen, Waltham, MA, USA) to obtain cDNA. The quantitative real-time PCR (qPCR) step of the qRT-PCR protocol was performed with the cDNA as the template using a SYBR Premix Ex Taq kit (Takara, Dalian, China) in the QuantStudio 3 Real-Time PCR System (Thermo Fisher Scientific, Waltham, MA, USA). *ACTIN2* was amplified as an internal control, and mRNA levels were standardized to those of pathogenesis-related gene 1 (*PR1*) using specific primers [31].

### 4.11. Statistical Analysis

Statistical significance was determined through a paired-sample *t*-test (Student’s *t*-test) or one-way analysis of variance (Tukey’s test) analysis (SPSS version 19.0, USA). Data were shown as mean ± standard deviation (SD). At least three biological repetitions were conducted in each experiment.

## 5. Conclusions

**Figure 5:**
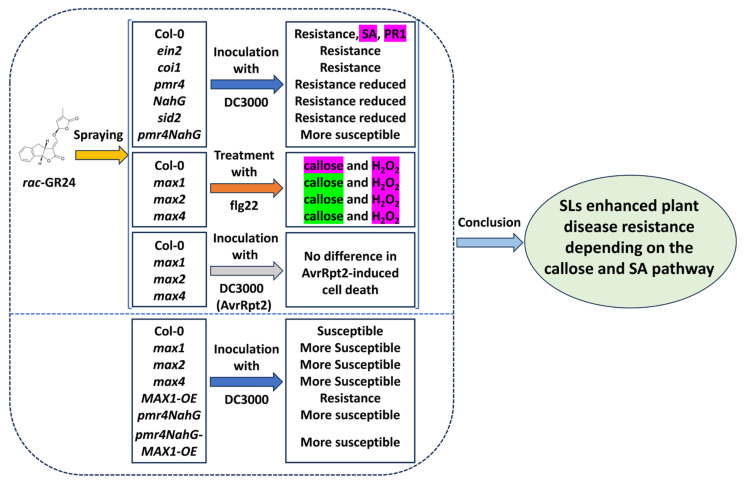
Flow chart and conclusion of SL-mediated disease resistance in Arabidopsis. In the upper part, three groups of plants were pretreated with *rac*-GR24 and then carried out with the corresponding treatment. In the lower part, a group of plants was inoculated with bacteria strain DC3000. Record the phenotype of each treatment. Metabolites highlighted in pink or green indicate an increase or decrease in their levels.

The results of the present study provide genetic evidence that clarifies the role of callose and SA in SL-mediated disease resistance (Figure 5). The callose synthase mutant *pmr4*, the SA-deficient *sid2* mutant, and the SA-deficient NahG-transgenic line reduced *rac*-GR24-induced resistance to DC3000. By contrast, the JA-signaling mutant *coi1* and the ET-signaling mutant *ein2* did not show altered *rac*-GR24-induced resistance. In particular, *pmr4*-NahG plants completely lost *rac*-GR24- and *MAX1*-induced resistance. Taken together, our results revealed that callose and SA are key determinants of SL-mediated disease resistance during *A. thaliana-P. syringae* interactions.

## Data Availability

The data and Supplementary Materials supporting the conclusions of this study are included within the article.

## Supplementary Materials

The following supporting information can be downloaded at https://www.mdpi.com/article/10.3390/plants13192766/s1, Figure S1: Identification of transgenic Col-0 plants; Figure S2: Colony morphology and diameter; Figure S3: Flg22-induced callose deposition; Figure S4: Identification of *MAX1*-transgenic *pmr4NahG* plants; Figure S5: JA and ET signaling are not required for SL-mediated resistance to DC3000; Figure S6: Pathogenicity analysis; Table S1: The primers used in identification of mutants/transgenes.
