# Supplementary material for: Callose and Salicylic Acid Are Key Determinants of Strigolactone-Mediated Disease Resistance in Arabidopsis
Source: Plants (Basel). 2024 Oct 2;13(19):2766. doi: 10.3390/plants13192766 (PMC11478789; doi:10.3390/plants13192766)
Supplement: Supplementary file 1 [file plants-13-02766-s001.zip › plants-3205121-supplementary.pdf]

Supplementary Materials

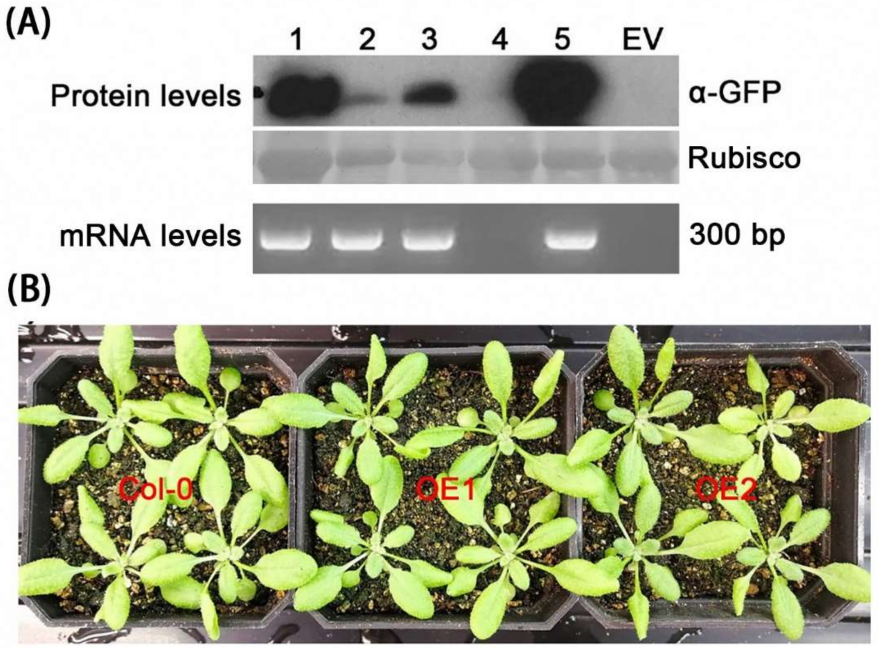

Figure S1. Identification of transgenic Col-0 plants. (A) Protein and mRNA levels in *GFP-MAX1* transgenic lines 1 to 5. EV, empty vector. Total proteins of leaves were detected with anti-GFP antibody. mRNA levels were quantitated by PCR (cDNA as template). Approximately 300 bp DNA fragment was amplified. The primers listed in Table S1. (B) Growth phenotype of plants, including Col-0 and transgenic lines OE1 (line 1) and OE2 (line 5).

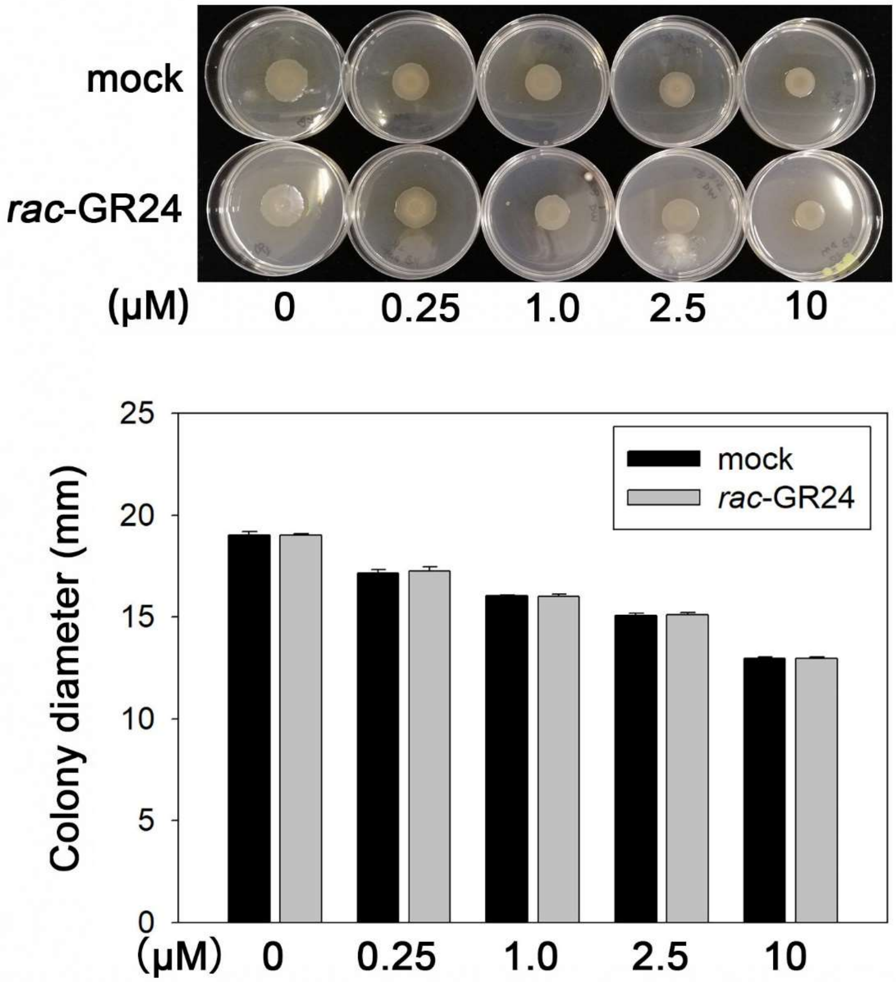

Figure S2. Colony morphology and diameter. The DC3000 strain grew to 48 hr at 28°C on KB agar medium contained various *rac*-GR24 concentrations (0, 0.25, 1.0, 2.5, and 10 μM). DMSO, separately 0, 0.175, 0.7, 17.5, and 7 mM, were used as the corresponding control.

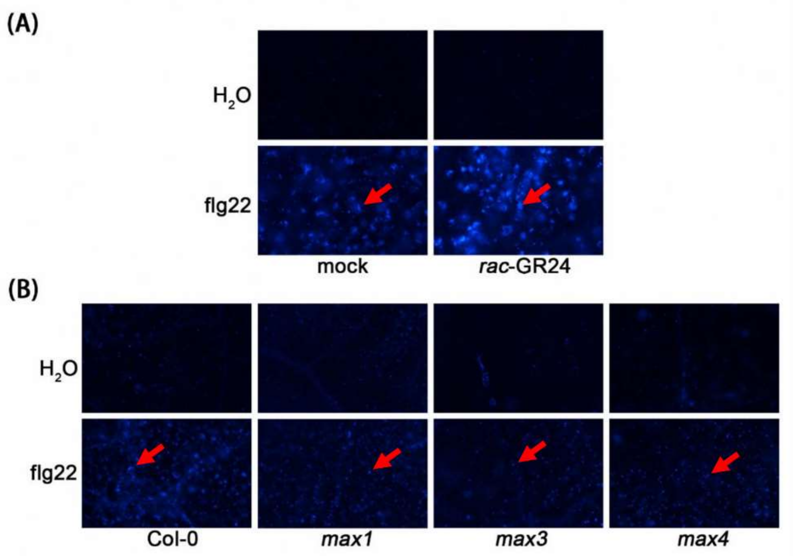

Figure S3. Flg22-induced callose deposition. (A) Callose fluorescence in leaves of Col-0 plants. (B) Callose fluorescence in leaves of Col-0 and SL biosynthesis mutants *max1*, *max3*, and *max4*. Leaves were injected with 1 μM flg22 for 24 h, and aniline blue staining was observed using a microscopy. Red arrow indicates the callose fluorescence.

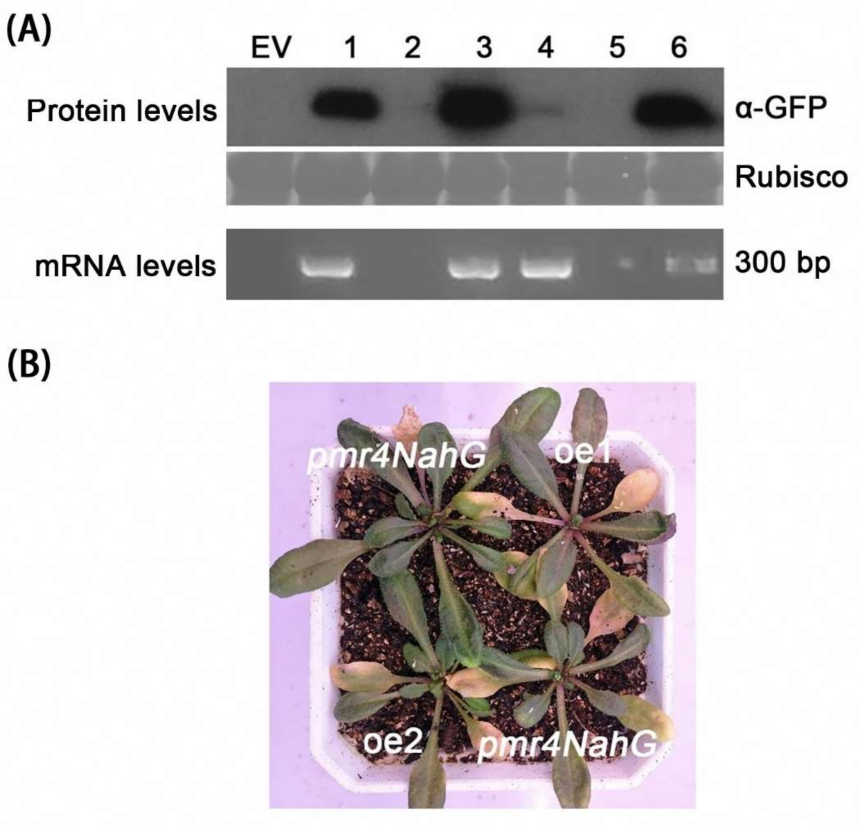

Figure S4. Identification of *MAX1*-transgenic *pmr4NahG* plants. (A) Protein and mRNA levels in *GFP-MAX1* transgenic lines 1 to 6. EV, empty vector. Total proteins of leaves were detected with anti-GFP antibody. mRNA levels were quantitated by PCR (cDNA as template). Approximately 300 bp DNA fragment was amplified. The primers listed in Table S1. (B) Growth phenotype of plants, including *pmr4NahG* and transgenic lines oe1 (line 1) and oe2 (line 3).

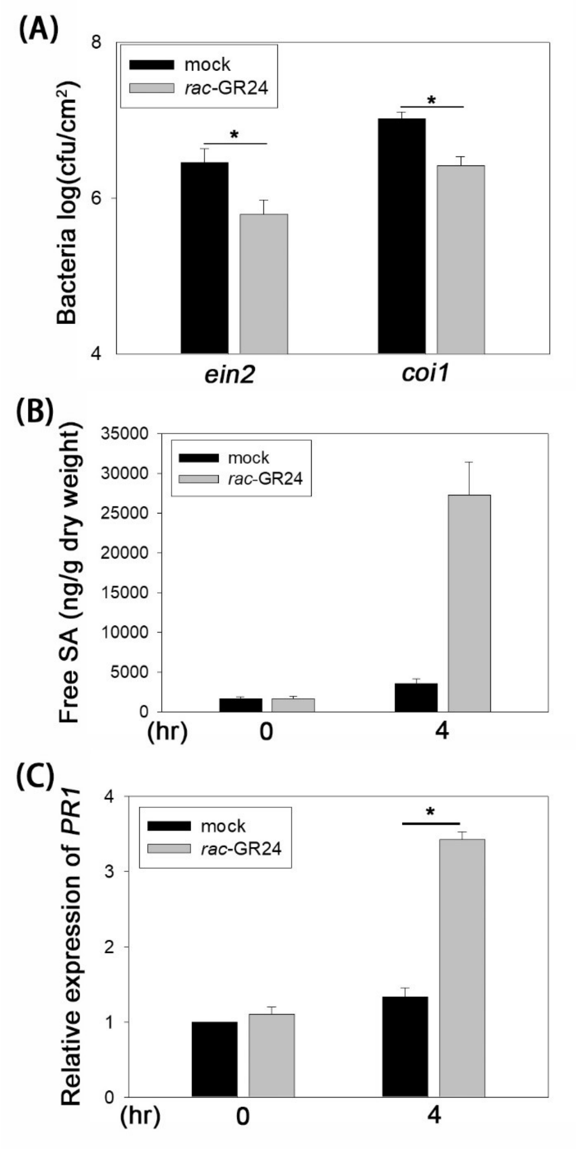

Figure S5. JA and ET signaling are not required for SL-mediated resistance to DC3000. (A) Pathogenicity analysis. Mutants *ein2* and *coi1* were sprayed with *rac*-GR24 and DMSO (as mock) before inoculation with DC3000. At 3 dpi, the number of bacteria in infected leaves was counted. Error bars, ± SD. *, *t*-test, $P < 0.05$. Similar results were obtained in three independent experiments. (B) *rac*-GR24 promotes induces free-SA content in *ein2* mutant. The mutant was treated with *rac*-GR24 and DMSO (as mock) for 4 hr, and free-SA contents in leaves were measured. Error bars, ± SD. Similar results

were obtained in another two independent experiments. (**C**) *rac*-GR24 promotes SA-dependent gene expression of *PR1*. Error bars, ± SD. *, *t*-test, $P < 0.01$. Similar results were obtained using a second set of samples.

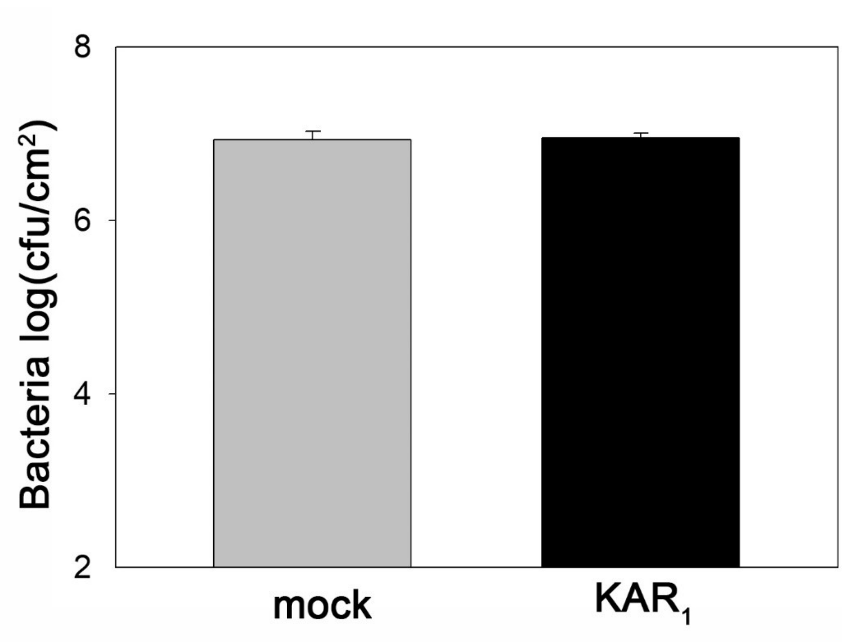

Figure S6. Pathogenicity analysis. Col-0 plants were sprayed with KAR_1 (one type of karrikins) and DMSO (as mock). Treated leaves were inoculated with DC3000. At 3 dpi, the number of bacteria in infected leaves was counted. Similar results were obtained in three independent experiments.

Table S1. The primers used in identification of mutants/transgenes

Mutants/Transgenes	Forward primers	Reverse primers
max1	GGCCATCTCTTATGGTT	GTAGGGATAAGACTTGT
max3	ATTGAATCTGGATCGTT	GTATCAACTTGAATTCCG
max4	GAAGGTGGAAGGTGAG	AGCGTCGGATTCAAGGA
pmr4	AGTCCGTGGAAGCATG	TTAGATAGCTGTTCAGTT
NahG	CAGGTACAGCTGTTCG	GATGCAGGCTGTCGACC
GFP-MAX1 transgenes	CAACCACTACCTGAGCAC	CATTGCATACGGACCAGG
